# Friction Performance of Aged T-BFRP Composite for Bearing Applications

**DOI:** 10.3390/polym10101066

**Published:** 2018-09-25

**Authors:** Umar Nirmal

**Affiliations:** Centre of Advanced Mechanical and Green Technology, Faculty of Engineering and Technology, Multimedia University, Jalan Ayer Keroh Lama, Melaka 75450, Malaysia; nirmal@mmu.edu.my or nirmal288@zoho.com; Tel.: +606-252-3023 or +6012-944-3261

**Keywords:** friction, natural fibre, aging, polyester, composite, thermo-mechanical loading

## Abstract

The current work is an attempt to reduce friction coefficient of the treated betelnut fibre reinforced polyester (T-BFRP) composites by aging them in twelve different solutions with different kinematic viscosities. The test will be performed on a pin on disc (POD) wear test rig using different applied loads (5–30 N), different sliding distances (0–6.72 km) at sliding speed of 2.8 m/s subjected to a smooth stainless steel counterface (AISI-304). Different orientations of the fibre mats such as anti-parallel (AP) and parallel (P) orientations subjected to the rotating counterface will be considered. The worn surfaces were examined through optical microscopy imaging and it was found that the aged specimens had significantly lower damages as compared to neat polyester (NP) and the unaged samples. Besides, P-O samples revealed lower friction coefficients as compared to AP-O, i.e., reduction was about 24.71%. Interestingly, aging solutions with lower kinematic viscosities revealed lower friction coefficients of the aged T-BFRP composites when compared to the ones aged in higher kinematic viscosities.

## 1. Introduction

In the field of *Tribology of Polymeric Composites*, the study of friction characteristics is crucial because materials with higher friction coefficient (µ) tend to have poor wear performance and vice versa [1]. As a matter of fact, different reinforcements in the polymeric composite directly influences the friction coefficient of that material/composite when it is subjected to sliding motion. Having that said, researchers, engineers and scientists work hand to hand to enhance the performance of natural fibres, a “greener” alternative to synthetic fibres for the use in polymeric composites in bearing applications.

Faissal and Mohamed [2] investigated the friction scale effect on drilling flax fibre composites. They revealed that the tribo-mechanical behavior of the drilling operation is affected by changing the tool coating at different levels. Hence, it could be said that the abrasive wear imposed by the flax fibre composites to the tool wear is low. This can promote high tool life span and save on costs. Previously, the participating author had investigated the effect on abrasiveness to process equipment using betelnut and glass fibres reinforced polyester composites [3]. The work revealed similar findings as reported in [2] where betelnut composite showed less damage on the cutting blade saw as compared to the glass fibre composite. This was due to the low density of betelnut fibres, which in return eased relative motion during the cutting process and caused low friction during the cutting process.

Previously, a critical comparative study was performed on betelnut fibres as an alternative to glass fibres reinforced thermoset composites [4]. The work revealed that treated betelnut fibre reinforced polyester (T-BFRP) composite showed improved mechanical properties by 1.16%, 17.39% and 4.92% in tensile, flexural and compression, respectively. Through tribological performance tests, T-BFRP composite showed superiority in wear for the dry and wet tests of about 98% and 90.8% while the friction coefficient was reduced by about 9.4% and 80% respectively. The interface temperature was lower by about 17% for T-BFRP composite subjected to dry test as compared to glass fibre composites. Scanning electron microscopy (SEM) analysis revealed that the brittle effects observed on glass fibres during the tribo test enhanced the material removal rate, which increased the thermo-mechanical effects on the rubbing zone. As such, evidence of adhesive to abrasive wear transition was observed when the glass fibre composite was subjected to the stainless steel counterface. On the contrary, T-BFRP composite formed a thin layer of shield (i.e., back film transfer) on its worn surface during the test, which assisted in lowering the material removal rate.

An attempt was made by the participating author in the year 2010 to study the effect on betelnut fibres treatment and contact conditions on frictional performance of polyester composites [5]. The frictional performance of T-BFRP composite are highly controlled by fibre mat orientations. As such, the presence of treated betelnut fibres in the matrix improved the frictional performance of polyester, especially when the fibre mats were orientated anti-parallel to the sliding direction, i.e., the average friction coefficient was reduced by 95%, respectively, under wet contact conditions compared to the dry. At dry contact conditions the friction coefficients for the T-BFRP composite were in the range of 0.4–0.7 for anti-parallel orientation (AP-O), 0.5–0.75 for parallel orientation (P-O) and 0.5–0.8 for normal orientation (N-O). Thus, the thermo-mechanical loading of the T-BFRP composite at dry contact conditions were relatively high as compared to the wet conditions. This resulted in an unnecessary material removal process of the composite at the rubbing zone that ruined the wear performance of the composite at dry contact conditions.

Having said that, the current work is an attempt to improve frictional performance of the T-BFRP composite at dry sliding conditions. The composite will be aged in 12 different solutions at a period of three years before performing the tribological tests. These types of solutions (i.e., 5W/40, 5W/50, 10W/30, 10W/40, 10W/50, 15W/50, 20W/50, SAE80, ATF D-3, ATF D-4 and R134a) were selected because they are commonly used in automotive vehicles. Therefore, the secondary goal of the current work is to propose a suitable T-BFRP composite for the use in automotive components subjected to bearing applications. The test will be conducted on a Pin on Disc (POD) tribological wear test rig using different contact conditions of the composites (AP-O and P-O) at different applied loads (5–30 N) in different sliding distances (0–6.72 km) and at fixed sliding velocity of 2.8 m/s subjected to a smooth stainless steel counterface. The friction will be compared with the controlled test specimen, aged neat polyester and the wear modes will be investigated using optical microscopy imaging.

## 2. Methodology

### 2.1. Preparation of the Fibres

**Figure 1:**
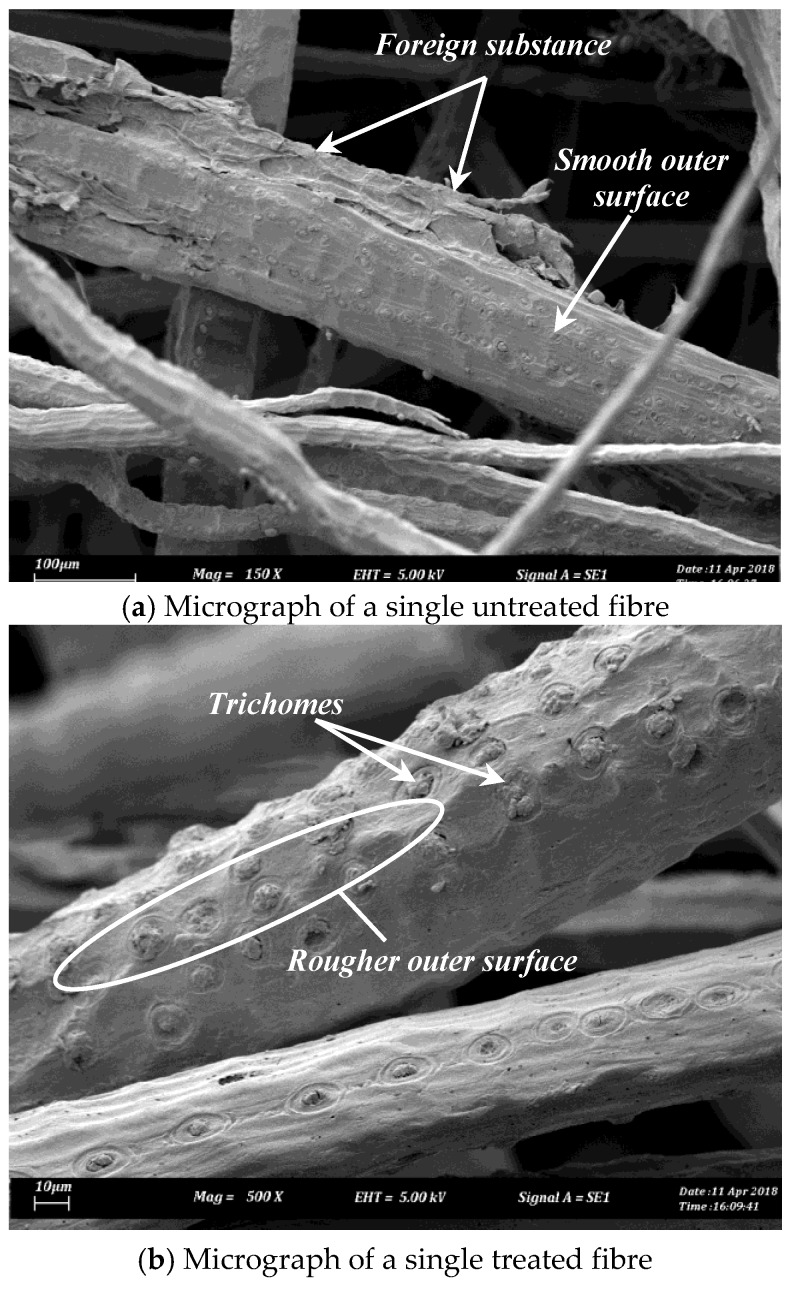
SEM micrographs of single betelnut fibre before and after treatment.

**Figure 2:**
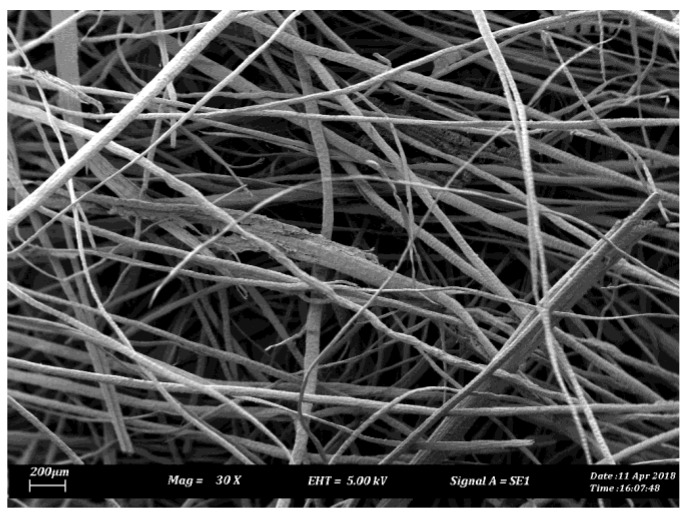
SEM micrograph of treated fibre mat.

Raw betelnut fruits were obtained from Alor Setar, Kedah, Malaysia. 6% NaOH was used to treat the fibres (Chemibond Enterprise Sdn. Bhd, Selangor, Malaysia). More information on the steps of fibre preparation and treatment process can be found in [6]. Figure 1a,b show scanning electron microscopy (SEM) micrographs of the single betelnut fibre before and after treatment. The micrographs were taken using a SEM machine (model: JEOL, JSM 840, Melaka, Malaysia). Before taking the micrographs, the surface of the fibre was coated with a thin layer of gold using ion sputtering (model: JEOL, JFC-1600). All observations were performed at room temperature of 28 ± 5 °C and at humidity level of 80 ± 5%. From Figure 1b, very rough fibre surface can be seen; rougher than in Figure 1a. This rough surface is attributed to the presence of *trichomes*. In previous works [4,7,8], it was discovered that the *trichomes* had served a purpose in increasing the surface wettability of the fibre in the resin matrix, which assisted in preventing fibre pull-out. To the best of literature knowledge, there is no single natural fibre reported having such fibre surface, which make betelnut fibres a unique candidate for reinforcement in polymeric composite. Subsequently, the fibre mats were then cut into the dimensions of the composite fabrication mold. Figure 2 shows a micrograph of a randomly oriented treated betelnut fibre mat. The average distance of the fibres in the mat was determined to be 83 ± 5 μm, which was computed using scaling method (Figure 2). Table 1 summarizes some important properties of the treated betelnut fibre mat.

### 2.2. Preparation of the Test Specimens

For the current work, Butanox M-60 unsaturated polyester resin mixed with 1.5% of methyl ethyl ketone peroxide (MEKP) as catalyst was selected (Bonding Resources Sdn. Bhd. Melaka, Malaysia). A closed mold with a size of 100 mm × 100 mm × 10 mm was used for preparing the composite. The inner surfaces of the mold were sprayed with a thin layer of silicon as a release agent. Hand layup technique was adopted where the first layer of the composite was obtained by pouring the liquid polyester (mixed with 1.5 wt % hardener) into the mold. Subsequently, the prepared treated fibre mats were placed on the first layer of polyester. A steel roller was used to distribute the fibres uniformly and to release the air bubbles from the mixture. This procedure was repeated until a maximum thickness of 10 mm was achieved (13 layers of fibre mats and 14 layers of polyester resin). Then, the mold was closed with a thin steel plate; and a pressure of about 5 kPa was applied on the top of the mold’s steel plate to ensure that the bubbles were forced out. With the pressure still being applied on the mold, the composite block was cured at room temperature (28 ± 2 °C) for 24 h. To ensure complete curing, the hardened composite was removed from the mold and post cured in an oven at 80 °C for 1 h. In a similar manner, neat polyester (NP) was also fabricated based on the steps mentioned above (i.e., without reinforcements).

**Figure 3:**
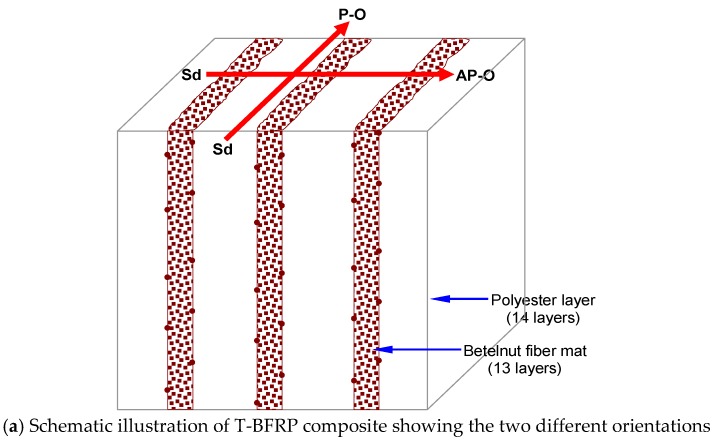

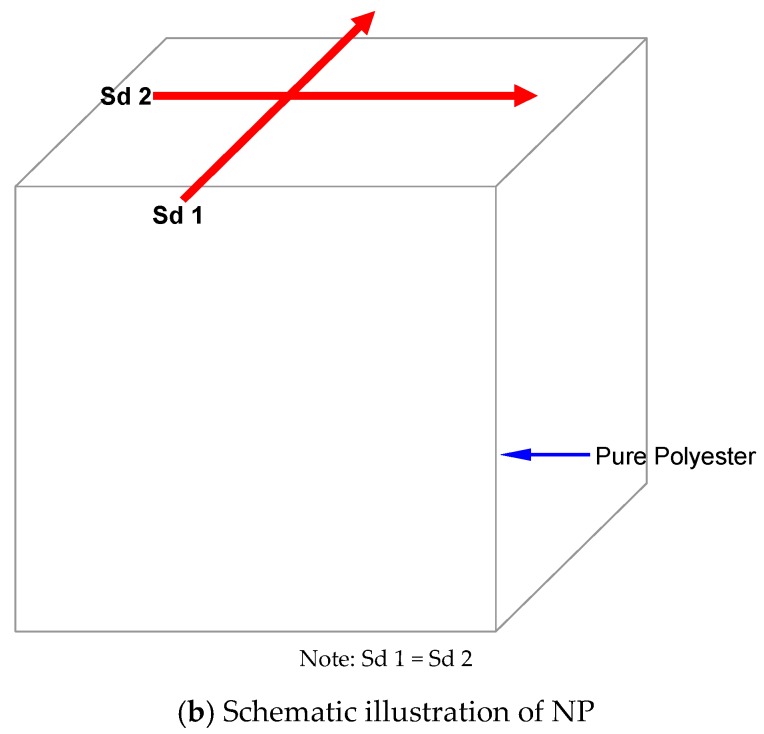
Corresponding schematic illustration of the specimen showing different fibre mat orientations with respect to the sliding direction.

For the tribological tests, test specimens with dimensions of 10 mm × 10 mm × 20 mm were cut out from the cured neat polyester (NP) and composite block using a Black and Decker jigsaw (model: CD301-B1, Melaka, Malaysia). A schematic view of the sample showing its orientation of fibre mats with respect to the sliding direction is displayed in Figure 3a while Figure 3b shows a schematic view of NP.

All test samples with dimension 10 mm × 10 mm × 20 mm were cleaned with a soft clean cloth to remove any debris, which was generated during the cutting process. Accordingly, the cleaned test samples were post cured in an oven for 5 h at 45 °C and their initial weights (i.e., before immersion) were recorded using a ±0.1 mg weight balance (model: Shimadzu AW120, Melaka, Malaysia). Subsequently, 5 to 6 specimens were immersed in different solutions with different kinematic viscosities for a period of 3 years at monitored room temperature (30 ± 5 °C). The kinematic viscosities of the solutions used are presented in Table 2.

**Figure 4:**
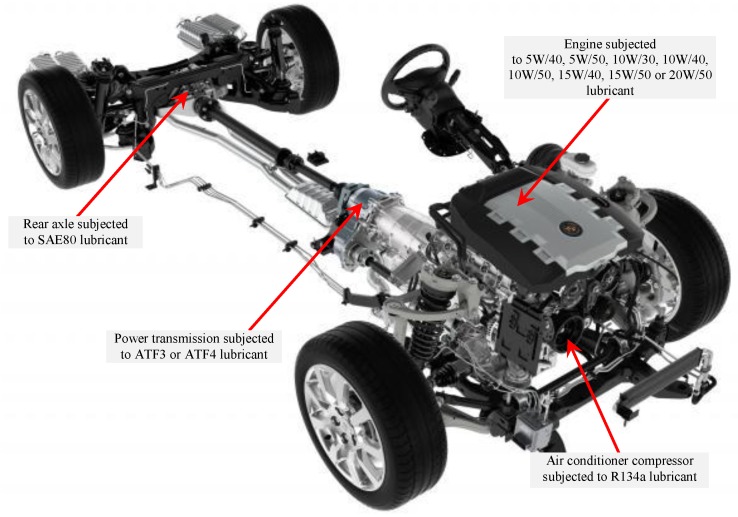
Typical locations of an automotive vehicle subjected to different types of lubricants.

For the current work, 12 types of lubricants with different kinematic viscosity were chosen as the aging solutions for the T-BFRP composite. This is mainly due to the fact that these lubricants are commonly used by the automotive vehicles and hence the current work is an attempt to explore the suitability of using the aged T-BFRP composite for tribological applications particularly in the automotive sector. Figure 4 illustrates the typical components of an automotive vehicle subjected to the different types of lubricants [9].

**Figure 5:**
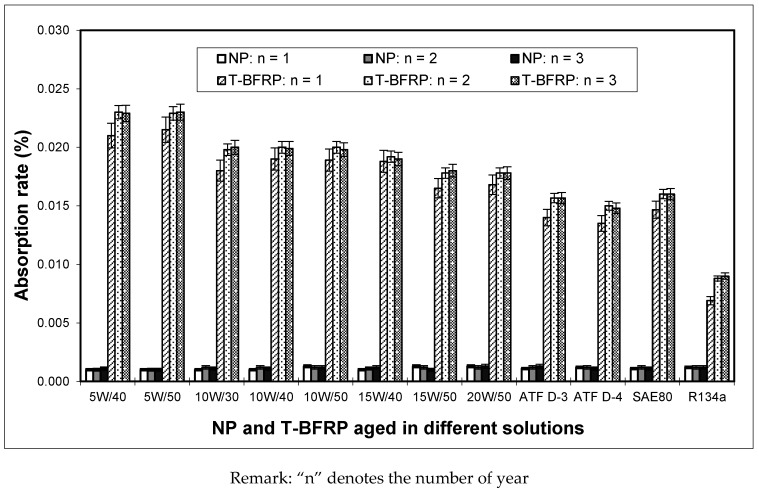
Absorption rate (%) of NP and T-BFRP composite at different immersed solutions for a period of three years.

After 3 years, all specimens were carefully taken out of their respective solutions and dried by means of wrapping them with tissue papers for 1 week at room temperature (30 ± 5 °C). The reason tissue papers were used is because they had excellent absorbing capabilities [14]. The tissue papers were then removed and all test specimens were post dried in a one way moisture flow ventilated oven (model: Panasonic NN-GS597A, Melaka, Malaysia) at 50 °C for one day. This type of oven was used to enable the trapped moisture/solution in the specimens to flow out from the oven’s chamber (i.e., through a one-way valve) during the drying process and at the same time preventing the surrounding moisture to enter into the oven’s chamber. The weights of all specimens were then recorded using a ±0.1 mg weight balance (model: Shimadzu AW120, Melaka, Malaysia). Next, the specimens were individually placed in an air tight container to prevent any moisture absorption by the samples prior to the tribo test. Silica gels were placed in the air tight containers to absorb any moisture present in the containers. Their corresponding absorption rates for a period of three years with respect to the different solutions are presented in Figure 5. From the Figure, it is noticed that the samples soaked in solutions with higher kinematic viscosities experienced low absorption rate while the samples soaked in solutions with lower kinematic viscosities experienced higher absorption rate. This is due to the ease of absorption by the betelnut fibres in the resin matrix at low kinematic viscosity, i.e., low fluid shear resistance. Hence, at higher viscosities, the fluid is “thicker” whereby the fibres find it hard to absorb during the soaking period. For the case of NP, Figure 5 illustrates that the absorption rate did not change for an aging period of 3 years. The average absorption values of the samples were in the range of 0.001–0.002% for all types of solutions. This slight absorption could have been due to the micro-pores on the NP samples, which trapped a small amount of solution during the aging process. On the other hand, it is interesting to note from Figure 5 that from n = 2 to n = 3, there was not any remarkable change in the absorption rate of the samples soaked in the different aging solutions. This could be due to the fact that the fibres had resisted further absorption into its cell wall. With respect to the obtained results, it justifies the current work to set the aging period at three years.

### 2.3. Experimental Procedure

**Figure 6:**
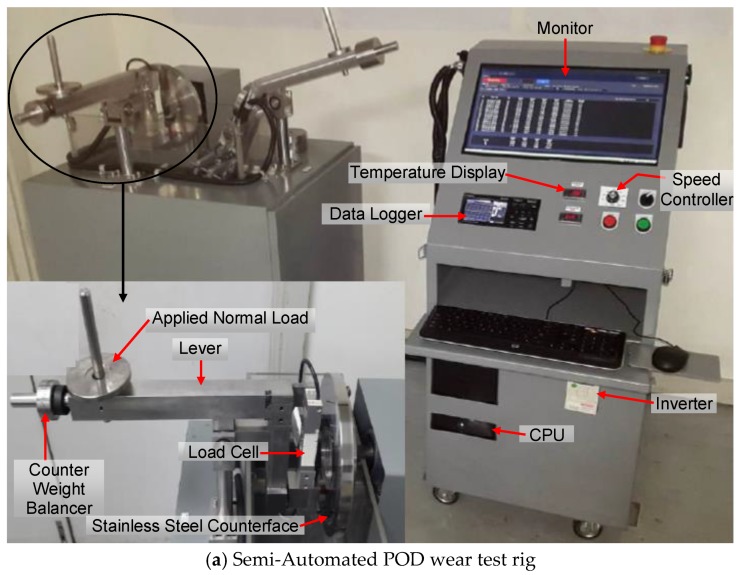

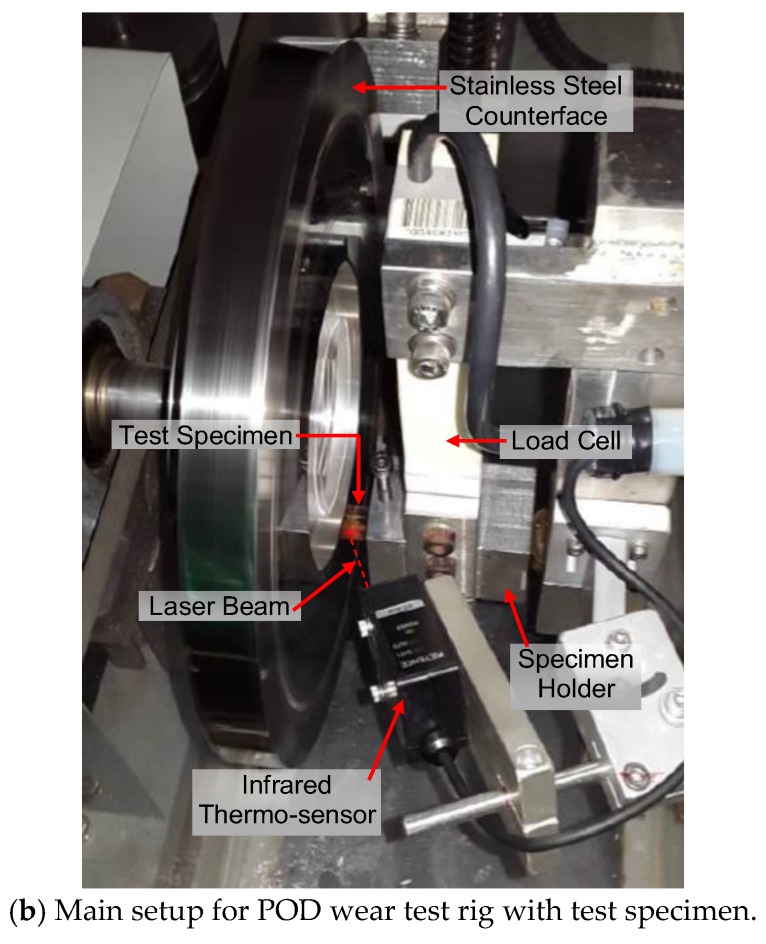
POD wear test rig and its important components.

Dry adhesive sliding wear test of the T-BFRP composite was conducted on an indigenous POD tribo test machine, Figure 6. A load cell (model: Accutec B6N-50, Melaka, Malaysia) was adopted in proper position to measure frictional forces while a data logger (model: OMRON ZR-RX25, Melaka, Malaysia) was integrated directly to the load cell to capture the frictional readings (refer Figure 6a). The friction coefficient (µ) was determined by dividing the measured frictional force recorded by the data logger with the applied normal used [15].

An ultra-compact digital infrared temperature sensor (model: KEYENCE FT-W Series, Melaka, Malaysia) was fixed and pointed at the interface of the test specimen and the stainless steel counterface of type AISI-304 with Brinell Hardness value of 123 to measure the interface temperature during the test, Figure 6b. The temperature sensor was able to capture temperatures in the range −50–1400 °C. It is to be mentioned here that all values of friction forces and temperature data were automatically captured via an integrated 10 channel data logger (model: OMRON ZR-RX25, Melaka, Malaysia) which was directly connected to a computer. The data logger was capable of capturing input parameters at any given time interval. For the current work, the time interval for friction and temperature recordings was set at 1 min.

Before starting the dry adhesive test, the T-BFRP composite test specimen was loaded onto the specimen holder of the POD machine while an abrasive paper grade 800 was placed between the counterface and the test specimen followed by an applied normal load of $F_{N}$ = 20 N to the POD machine. Prior to this, the counterface was turned manually by hand specifically to achieve proper intimate contact of the test specimen against the counterface. When this was achieved, the procedure was repeated for the same test specimen using abrasive papers grade 1000 and grade 2000 respectively. This was to minimize mechanical interlocking of the test specimen against the counterface during the start of the test. Upon completion, the test specimen was taken out, cleaned with a soft clean cloth and weighed using a ±0.1 mg weight balance (model: Shimadzu AW120) before starting the experiment. All specimens were individually kept in a labeled “air-tight” plastic container illustrating the type of test parameters to which they were subjected, i.e., applied normal load, sliding distance, fibre mat orientations and aging conditions.

Concerning the stainless steel counterface, it was polished with abrasive papers starting with grades 200, 500, 1000 followed by 2000. Surface roughness average (*R*a) of the stainless steel counterface after the polishing process was 0.052 ± 0.02 µm. During polishing, the counterface was cleaned with a clean cloth using acetone. To avoid conflict in friction readings generated during the test, which might be influenced from the thin layer of acetone produced during the counterface cleaning process, the whole counterface was wiped with a wet cloth and dried at room temperature (28 ± 2 °C) before each experiment. After the test, the specimens were cleaned with a soft dry cloth. This was particularly to remove the generated wear debris on the specimen surfaces.

The tests were performed at a controlled room temperature (30 ± 5 °C) and at a humidity level of 80 ± 5%. Each test was repeated three times and the average of the measurements was determined (i.e., this was done mainly to meet the requirements of ASTM G99-05 standard [15]). It is to be noted here that the indigenous POD tribological machine has a relative error of about <5%. In other words, the POD machine has a repeatability accuracy of about 95% [16,17].

### 2.4. Examination of the Worn Surfaces: Photo Micrographs Analysis

A NK Metallurgical microscope (model: MT 7100, Melaka, Malaysia) was used to analyze the worn surfaces morphology of the aged NP and T-BFRP composite for AP and P orientations. All observations were performed at room temperature of 28 ± 5 °C and at humidity level of 80 ± 5%.

## 3. Results and Discussion

### 3.1. Friction Performance of T-BFRP Composite Aged in Various Solutions

**Figure 7:**
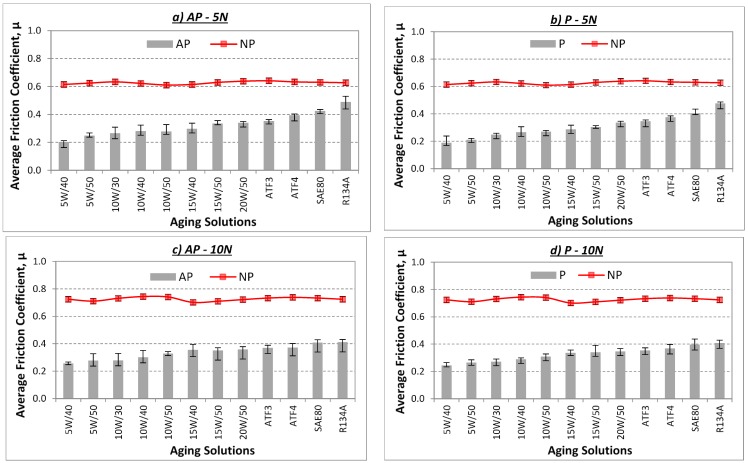

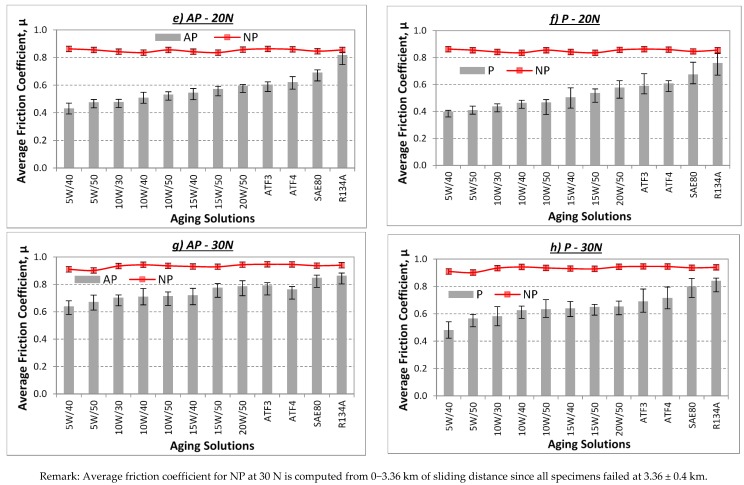
Average friction coefficient (µ) for the aged AP, P and NP test samples for different applied loads (5–30 N) and aging solutions subjected to a smooth stainless steel counterface at 2.8 m/s of sliding velocity and 6.72 km of sliding distance.

A visual examination of Figure 7 indicates a rise in friction coefficient for the aged AP, P and NP test samples when applied load increases. This phenomenon is normal since the contact pressure between the test specimen and counterface increases for higher applied loads. This causes high resistance of motion at the rubbing zone due to the high shear force exerted, noting the fact that the shear force is much higher for the aged NP test samples followed by P and AP test samples. In a work done by Zsidai and co-workers [18], it was reported that high values of friction between polymers and stainless steel material was possible when applied normal load increased. Thus, on the rough surface, i.e., NP test samples, deformation component is predominant while on the smooth surface, i.e., stainless steel counterface, adhesion component is predominant. This could explain the high values in friction coefficient of the aged NP test samples as compared to the aged AP and P test samples.

Since the aged AP and P test samples are governed by their anisotropic properties, the friction component differs in both test samples, i.e., fiction property is affected by the fibre mat orientations and fibre distribution at the rubbing zone [19]. It can be said that, for the aged P test samples, the tendency of fibre removal from the composite is much easier as compared to the aged AP test specimen. This is due to the parallel shear force, which could have contributed to the ease of relative motion between the test specimen and sliding counterface. Moreover, it is to be reminded here that the fibres had been aged in their respective solutions for three years, i.e., the betelnut fibre mats had absorbed a certain amount of solution (refer to Figure 4 for the absorption rate properties), which could contribute to the ease of sliding motion. However, this was not the case for the aged AP test sample since the sliding force was perpendicular to the betelnut fibre mats. Hence, the fibre experienced high shear resistance during the sliding wear test. More evidence of the aged AP, P and NP test samples after the test will be discussed later with the aid of the morphology of the worn surfaces.

Another observation is seen in Figure 7 where the average friction coefficient for the aged AP and P test samples increase for all the applied loads (5–30 N) when the kinematic viscosity of the aging solutions increase. Since viscosity of a fluid is a measure of its resistance to its gradual deformation by either shear stress or tensile stress, it corresponds to the concept of “liquid thickness” [20]. Hence, at higher “liquid thickness”, i.e., high kinematic viscosity, resistance of relative motion increases, which causes high friction coefficient between the T-BFRP composite and sliding counterface. In other words, it can be said that the absorption rates of the betelnut fibre mats in the different aging solutions do not contribute to the high values of friction coefficients but rather the kinematic viscosity itself is the governing factor. This indeed could explain the rise in friction coefficient for the aged AP and P test samples for the different applied loads when the kinematic viscosity of the aging solution increases. However, for the aged NP test samples, there was no change of friction coefficient for the different types of aging solutions used, i.e., the absorptivity rate by the NP samples was constant throughout the aging period of three years (refer to Figure 4).

**Figure 8:**
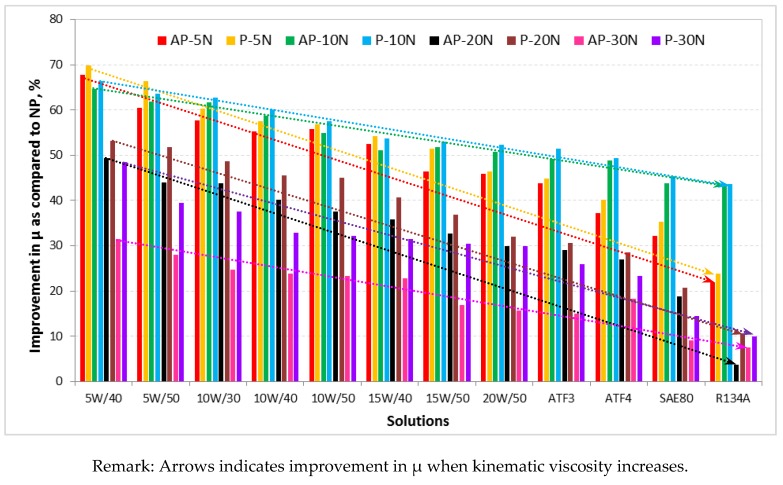
Improvement in percentage of µ for AP and P test samples as compared to NP subjected to the different aging solutions for different applied loads (5–30 N) at counterface sliding velocity of 2.8 m/s and 6.72 km of sliding distance.

The summary of friction coefficient improvement in percentage of the aged AP and P test samples as compared to the aged NP test samples is shown in Figure 8. From the figure, it can be seen that when the kinematic viscosity of the aging solutions increases, the improvement in friction coefficient gradually drops due to the high shear resistance incurred by the test specimen contacting surfaces against the smooth sliding stainless steel counterface.

### 3.2. Temperature Performance of T-BFRP Composite Aged in Various Solutions

**Figure 9:**
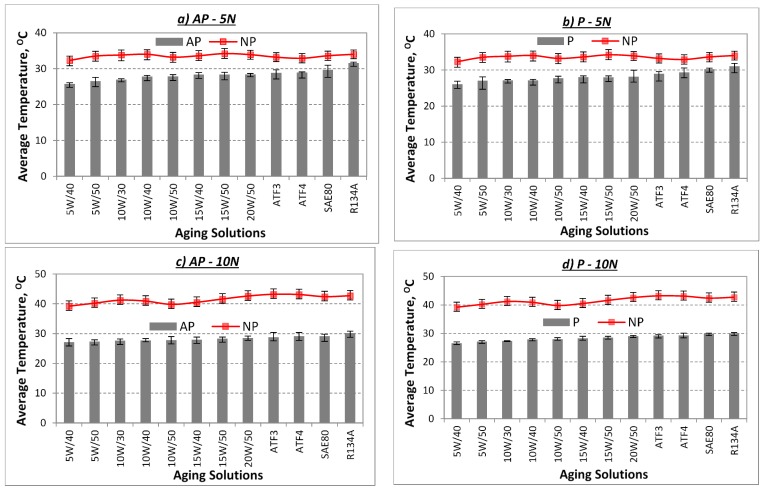

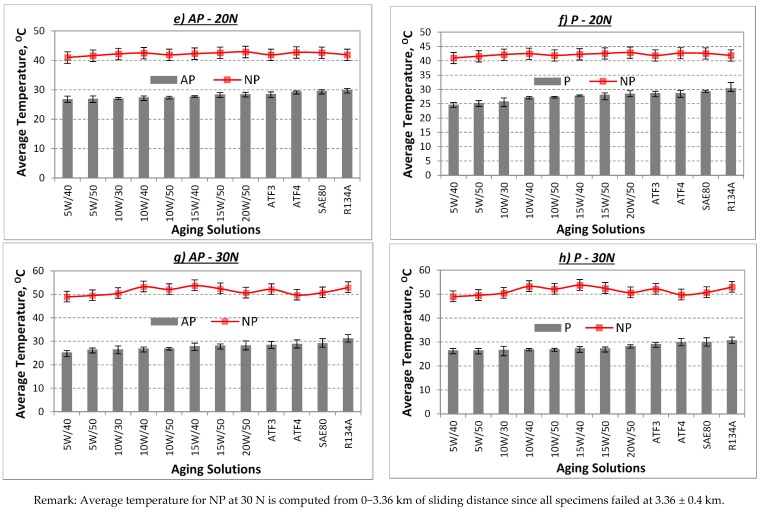
Average temperatures (°C) for the aged AP, P and NP test samples for different applied loads (5–30 N) and aging solutions subjected to a smooth stainless steel counterface at sliding velocity of 2.8 m/s and 6.72 km of sliding distance.

**Figure 10:**
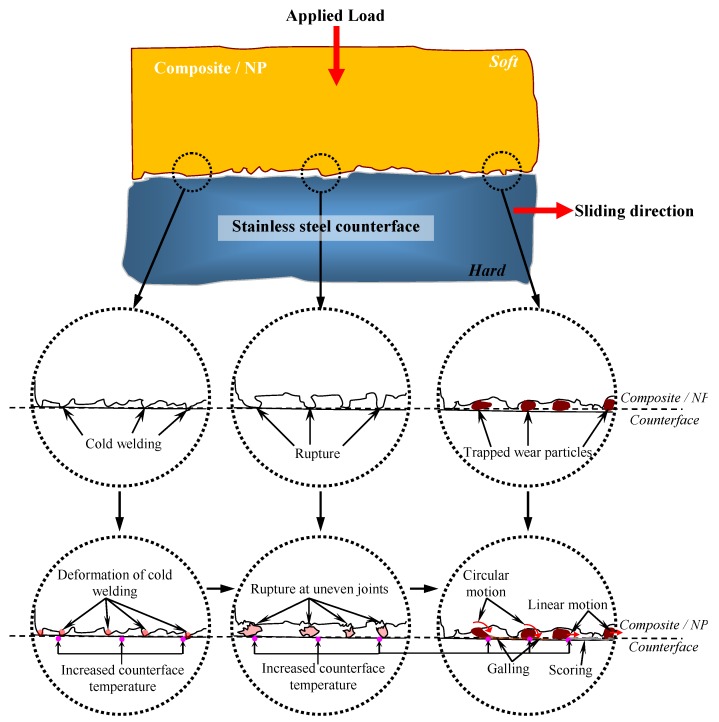
Schematic description of a typical thermo-mechanical loading process during a sliding wear test which involved two materials with different hardness values.

Average interface temperatures for the aged AP and P test samples are presented in Figure 9 respectively. The figure also shows the temperature profiles associated with their minimum and maximum values for the aged NP test samples in different aging solutions and under different applied loads. Generally, it is widely understood that when applied load increases during a typical sliding wear test between a composite/counterface, the interface temperature rises [21,22,23,24,25,26]. This is because, when the test specimen is subjected to the counterface before the sliding wear starts, “cold welding” is formed. When sliding starts, the “cold welding” tends to “deform” and “rupture”, which can contribute to the increase in contact temperature. For higher applied loads, “deformation” and “rupture” rates become higher resulting in trapped wear particles between the contacting surface of the test specimen/counterface. These trapped wear particles could be in circular motion (i.e., causing “galling” on the test specimen worn surfaces) or linear motion (i.e., causing “scoring” on the test specimen worn surfaces) during the sliding wear test, which can further increase contact temperature. The explanation above is a typical description of a thermo-mechanical loading process during a sliding wear test, which involves two materials with different hardness value, i.e., composite and neat polyester is softer than stainless steel counterface [4]. The visualization on the explanation above is shown schematically in Figure 10.

A visual examination of Figure 9 indicates that the temperature for NP rises as applied load increases. For instance, the temperature range of the aged NP test samples was 30–35 °C for 5 N, 38–45 °C for 10 N, 40–45 °C for 20 N and 48–55 °C for 30 N of applied load. However, there was an obvious decrease in the interface temperature of the aged AP and P test samples as compared to the aged NP test samples. This is mainly due to the fact that reinforcing NP with betelnut fibres had lowered the thermo-mechanical loading during the sliding wear test. Meanwhile, it was observed that for the aged AP and P test samples under the same applied loads, there was no remarkable change in the average temperature readings, noting that the temperature readings slightly increased when the kinematic viscosity of the aging solutions increased. Arguably, it can be said that at high kinematic viscosity, i.e., high shear resistance of the sliding surfaces, the relative motion incurred by the test samples was much higher than low kinematic viscosity, i.e., low shear resistance of the sliding surfaces. This can increase interface temperature since the friction component of relative motion of the composite/counterface is high; refer to Figure 7.

**Figure 11:**
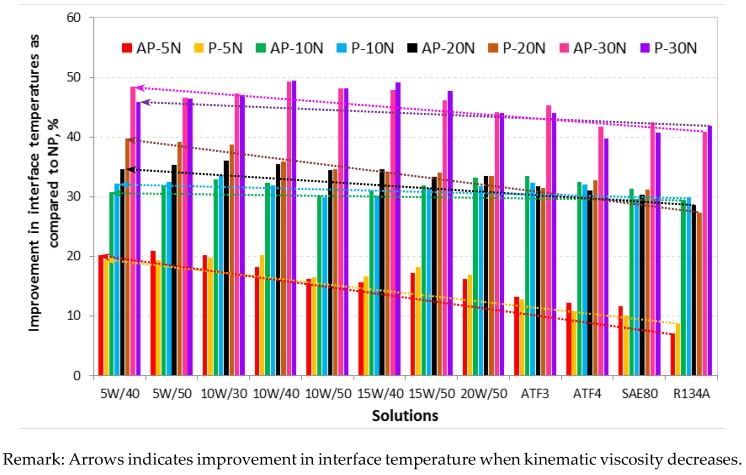
Improvement in percentage of interface temperature for AP and P test samples as compared to NP subjected to the different aging solutions for different applied loads (5–30 N) at counterface sliding velocity of 2.8 m/s and 6.72 km of sliding distance.

The summary of improvement in interface temperature in percentage for the aged AP and P test samples as compared to the aged NP test samples for the different aging solutions for different applied loads subjected to a counterface velocity of 2.83 m/s is shown in Figure 11 respectively. From the figure, it can be said that there was a significant amount of reduction of interface temperature (i.e., nearly 50% reduction) when NP was reinforced with betelnut fibres. Moreover, the figure also depicts that when the kinematic viscosity of the aging solution decreases, the reduction of interface temperature is far better than that at high kinematic viscosity (i.e., refer to Table 2 for the kinematic viscosity for the different types of aging solutions).

### 3.3. Morphology Analysis of the Worn Samples

**Figure 12:**
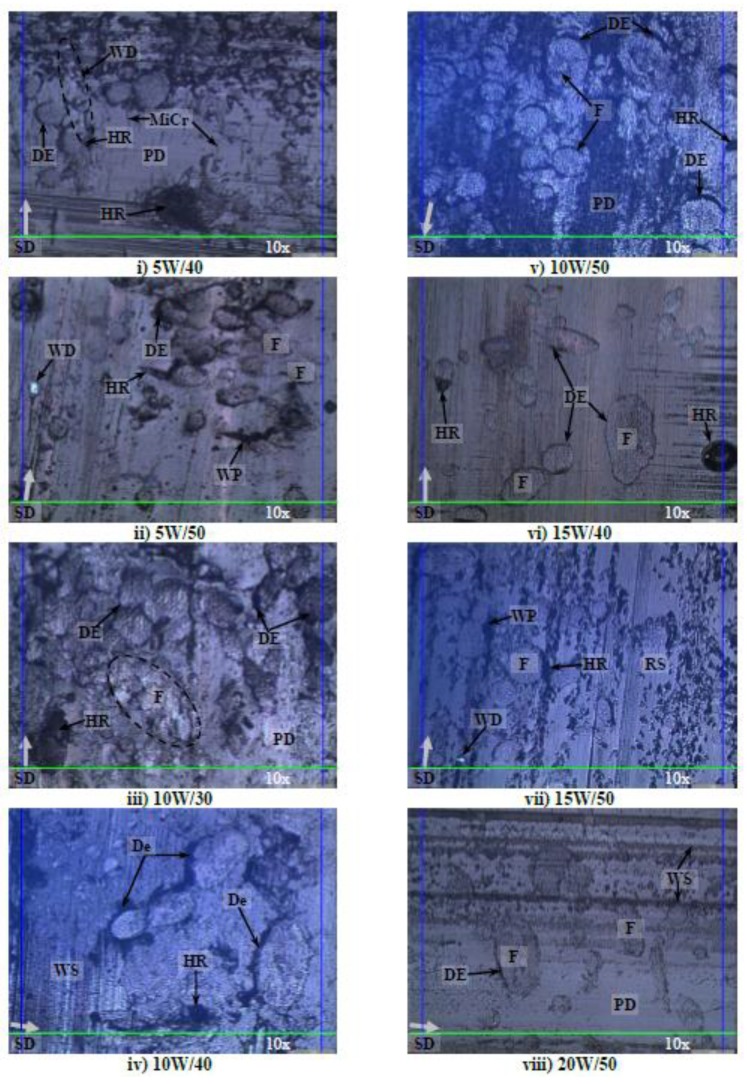

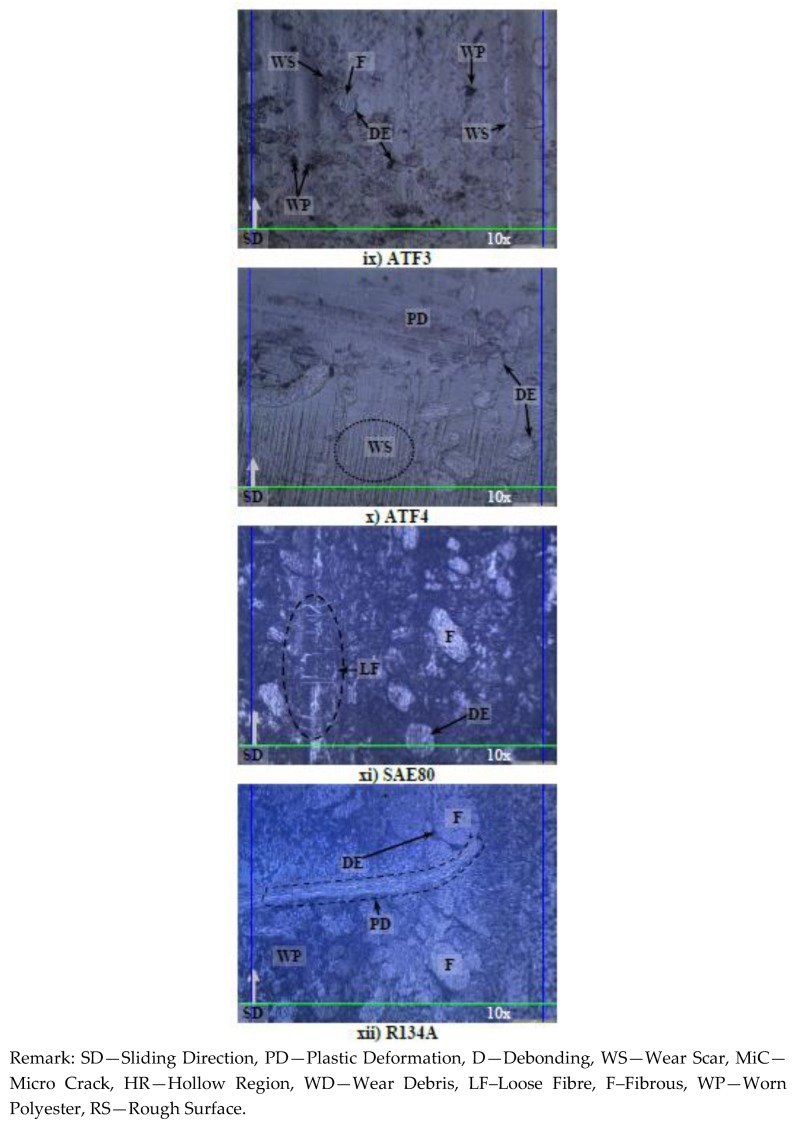
Photo micrographs of the worn samples for AP in different aging conditions at 30 N of applied normal load subjected to a smooth stainless steel counterface at sliding velocity of 2.8 m/s at 6.72 km of sliding distance.

**Figure 13:**
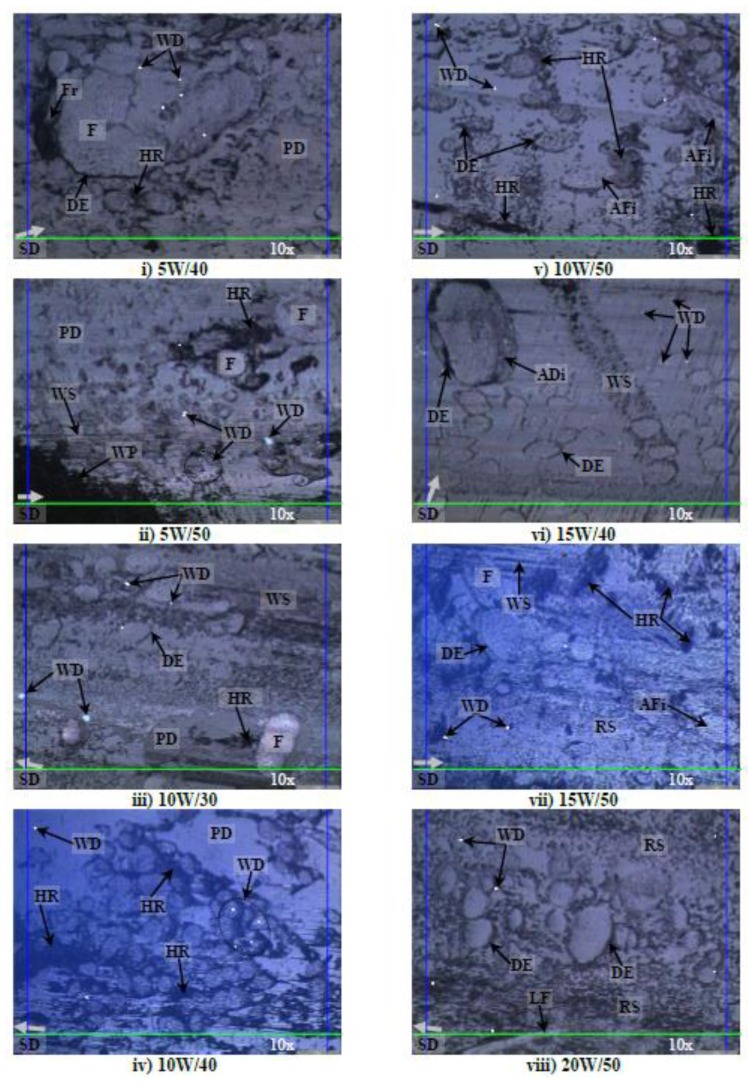

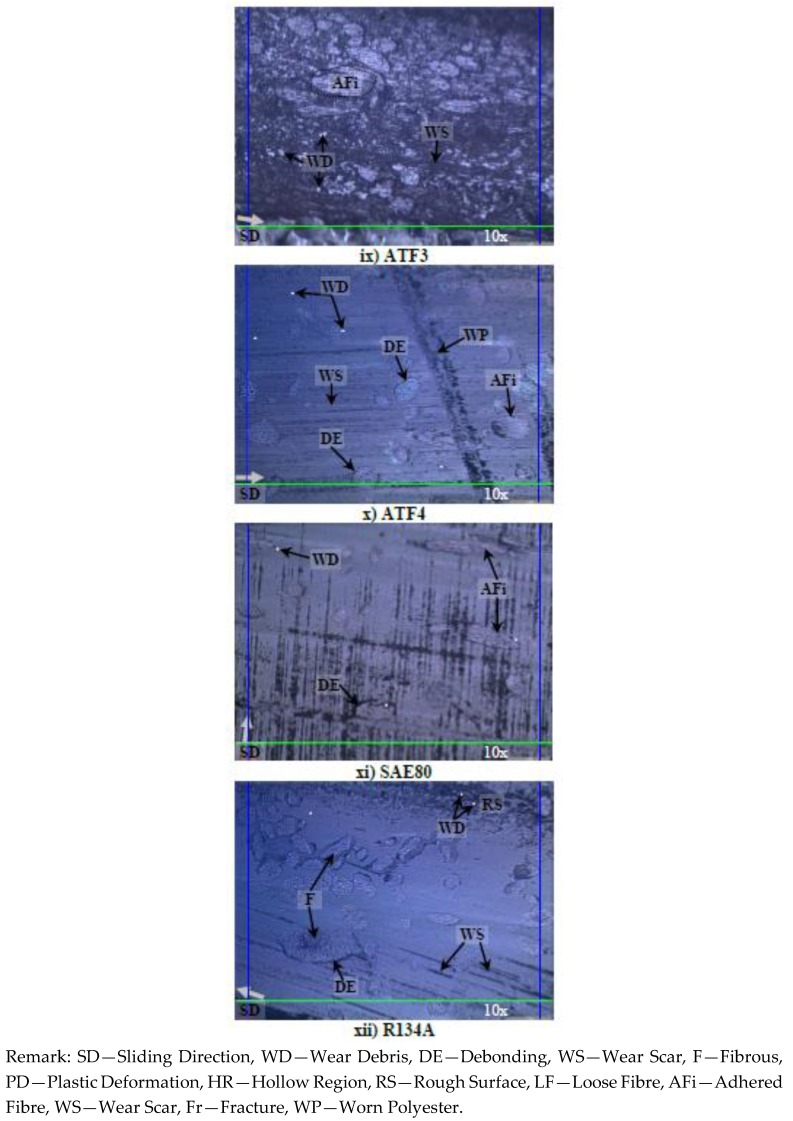
Photo micrographs of the worn samples for T-BFRP composite at P orientation in different aging conditions at 30 N of applied normal load subjected to a smooth stainless steel counterface at sliding velocity of 2.8 m/s at 6.72 km of sliding distance.

**Figure 14:**
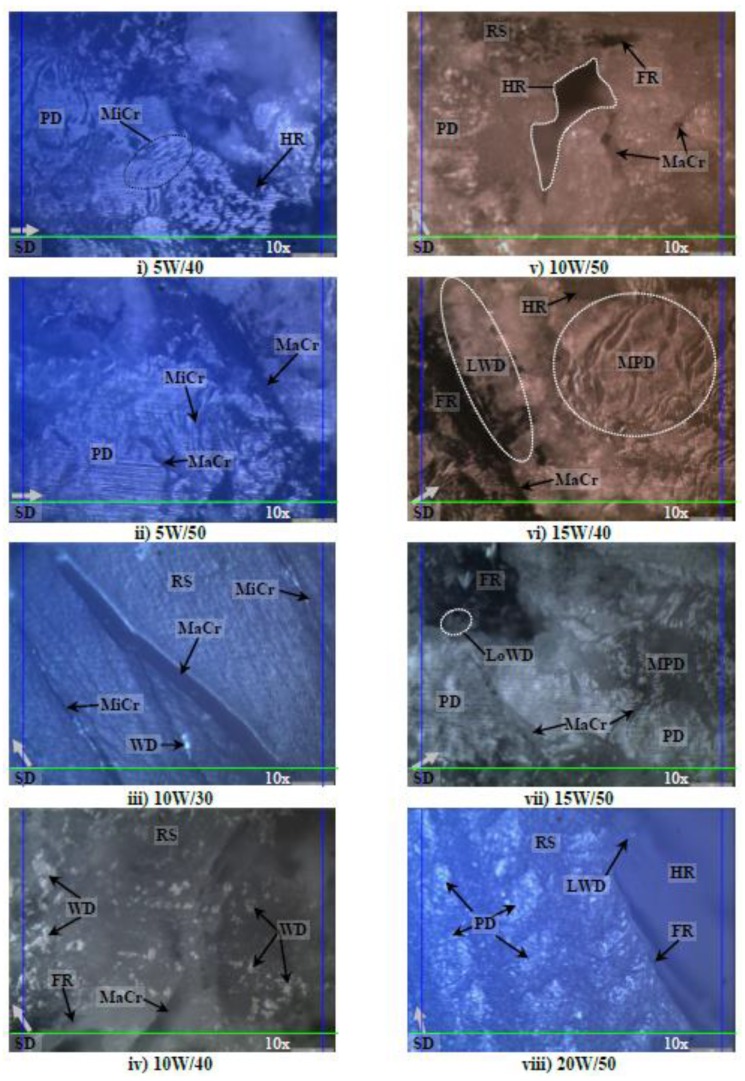

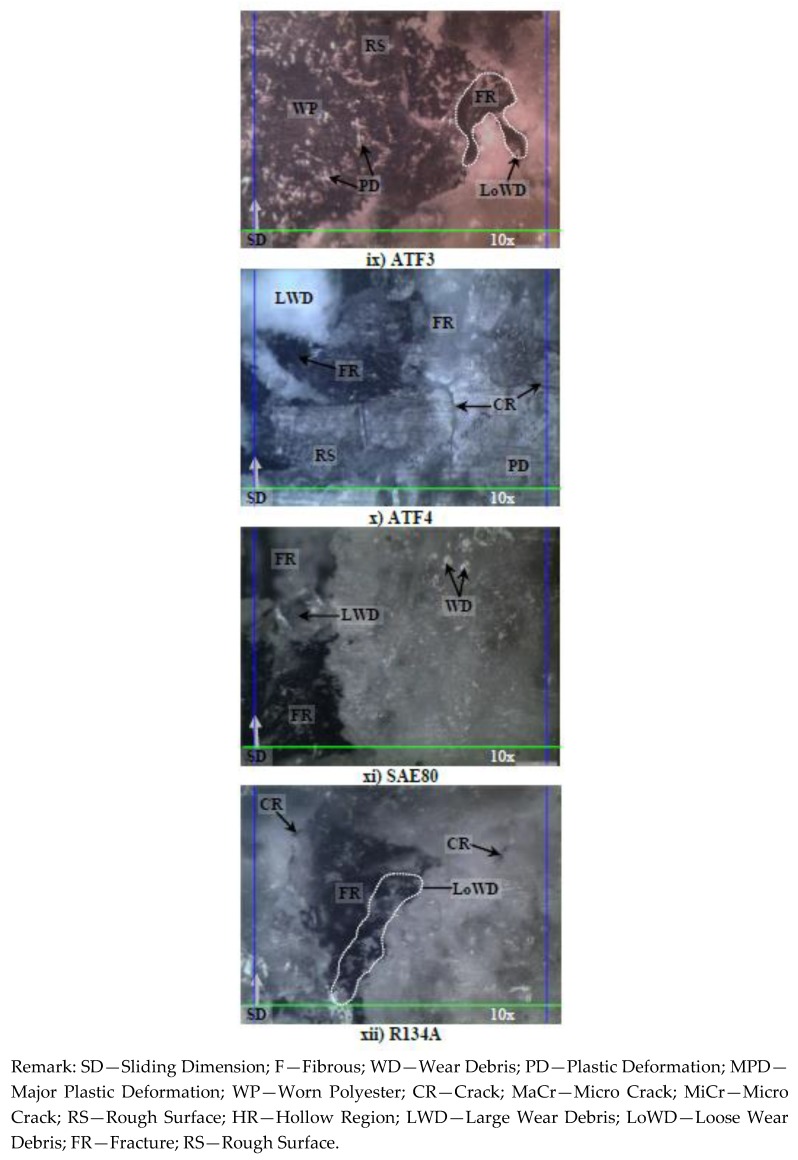
Photo micrographs NP in different aging conditions at 30 N of applied normal load subjected to a smooth stainless steel counterface at sliding velocity of 2.8 m/s at 3.36 ± 0.4 km of sliding distance.

Photo micrographs of the AP, P and NP worn samples in different aging conditions at 30 N of applied normal load subjected to a smooth stainless counterface at 2.8 m/s of sliding velocity and 6.72 km of sliding distance is presented in Figure 12, Figure 13 and Figure 14 respectively.

Logically, when contact pressure increases between the contacting surfaces of the test specimen and rotating counterface, higher material removal process of the composite is expected, i.e., high values of Ws. For the current work, high absorption rate by the treated betelnut fibres aged in their respective aging solutions is another governing factor to the different wear modes of the worn samples. For instance, Figure 12i–vi indicates that there were signs of hollow regions assisted with high amount of fibre debonding, i.e., labelled as “De” on the worn samples, against the resinous region. In other words, when absorption rate of the betelnut fibres is high, surface wettability of the fibre against the resin matrix decreases resulting in the fibre adhering parallel to the sliding direction. This results in a gap forming between the fibre and the resin matrix. This gap is crucial where it may turn to hollow regions (HR) due to the complete removal of the fibre during the sliding process. The wear modes were quite severe in Figure 12i–iii where there was a high amount of plastic deformation associated with micro cracks and micro fracture on the worn samples. Figure 12vii–x depicts that the wear modes of the worn samples were less significant when the absorption rate of the fibres reduced. Among the predominant wear modes were minor fibre debonding as the swelling rate of the fibres reduced (i.e., low absorption rate), formation of wear scars parallel to the sliding direction associated with mild plastic deformation in the resinous region. When the AP test samples were aged in SAE 80 and R134A solutions, the wear modes on the worn samples seems to be much less, i.e., low absorption by the fibres due to high kinematic viscosity of the aging solutions, see Figure 12xi–xii. Sign of loose fibre on the worn surfaces was at the minimal followed by low amount of fibre debonding against the resin matrix. The cross section of the fibre appeared to be in good shape indicating the ability of the fibre to carry the applied load well during the sliding process.

Figure 13 illustrates the photo micrographs of the worn samples for T-BFRP composite at P orientation in different aging conditions at 30 N of applied normal load subjected to a smooth stainless steel counterface at 2.8 m/s of sliding velocity at 6.72 km of sliding distance. An overview of the images of the worn samples indicates that the aged betelnut fibres was adhered parallel to the sliding direction of the counterface. Besides, the tendency of fibre pull-out for P orientation was high as there was evidence of hollow regions on most of the worn samples in different aging conditions. Moreover, there was evidence of scattered wear debris on all the worn sample images (i.e., Figure 13i–xii), which could be due to the ease of fibre pull-out at high applied load; 30 N. The damages were severe as depicted by Figure 13i as there were signs of fracture at the resinous regions associated with the evidence of worn polyester. It is understood that when the kinematic viscosity of the aging solution is low, the absorption rate by the fibres is high. This worsens surface wettability of the fibre against the resin matrix, which can contribute to high material removal process at high contact pressure, i.e., high values of Ws as compared to AP samples at 30 N of applied loads as seen in Figure 13j,k. Interestingly, it is to be noted that friction coefficients for aged P samples at 30 N of applied load was slightly lower than the aged AP samples, i.e., P_µ @ 30 N_: 0.5–0.82 while AP_µ @ 30 N_: 0.62–0.86 respectively (refer to Figure 7g,h). This could be clarified through the current worn images of the aged P test samples where the aged fibres could have acted as a “solid lubricant” during the sliding wear process thereby contributing to the ease of relative motion by the composite/counterface in P orientation. Remarkably, there was no sign of fibre tearing in the rubbing zone since most of the fibre cross section appeared to be in good shape as seen in Figure 13i–xii.

Figure 14 shows the photo micrographs of the NP worn samples in different aging conditions at 30 N of applied load subjected to a smooth stainless steel counterface at 2.8 m/s of sliding velocity and 3.36 ± 0.4 km of sliding distance. It is to be reminded here that all the NP test samples failed at approximately 3.36 ± 0.4 km of sliding distance (i.e., their corresponding worn surfaces are displayed in Figure 14i-xii respectively). Due to the absence of reinforcement, the pure polyester had thermally deformed (i.e., thermo-mechanical loading) leaving high amount of plastic deformation on all the worn samples. Besides, there were signs of micro-cracks (refer Figure 14i–iii), which propagated during the sliding process causing macro-cracks (refer Figure 14iii–vii) and finally fractured the test specimens (refer Figure 14iv–xii). On the other hand, there was evidence of loose wear debris trapped at the fractured/hollow region on the worn samples; Figure 14vii, ix and xii. This could help clarify on the higher values of friction coefficient of NP at 30 N of applied load (refer Figure 7g,h) since these loose wear debris could have been a “third body” between the sliding surfaces of NP/counterface. Hence, the wear mode could have changed from purely adhesive to abrasive sliding wear.

Although there was signs of huge gaps between the fibre and resin matrix on the worn samples, there was no evidence of complete fibre pull-out but rather partial fibre detachment from the resin matrix, i.e., sign of hollow regions on the worn samples as depicted in Figure 12i–vii. This partial detachment of fibre could have been due to the unique characteristics of betelnut fibre surfaces where the “*trichomes*” played a crucial role to firmly interlock the core of the fibre well in the resin matrix thereby preventing complete fibre pull-out. It is to be noted here that, if there was a sign of complete fibre pull-out, the resinous region will fracture due to insufficient reinforcement by the betelnut fibres; this was the case for AP test samples subjected to 30 N of applied load.

### 3.4. Comparison with Previous Works on Friction Performance of Polymeric Composites

Finally, it is interesting to compare the current results with available reported works on polyester composites based on synthetic and unaged betelnut fibres subjected to treated and non-treated conditions. Table 3 summarizes the friction coefficient and temperature results for the different types of composites under different operating conditions.

On the friction performance of the aged T-BFRP as compared to other published works, different values of friction coefficient are observed in Table 3. For the current work, P_5W/40_, recorded the lowest friction coefficient (i.e., 0.4820 ± 0.05) due to the lowest kinematic viscosity of the aging solution and the ease of relative motion in P-O as compared to AP-O. Besides, the current work (i.e., P_5W/40_) demonstrates reduction in friction coefficient by 29%, 40%, 49% and 45% as compared to unaged T-BFRP_P-O_, Ut-BFRP_P-O_, NP and CSM-GFRP_P-O_ composites.

On the average interface temperatures between the composite/counterface, the current work demonstrates a significant drop in temperature readings as compared to NP, unaged betelnut fibre composites and CSM glass fibre composites. It is to be noted here that the average interface temperatures for the aged NP was recorded up to 3.36 km in sliding distance since all the test specimens had failed at 3.36 km due to major “thermo-mechanical” loading at 30 N of applied load noting the fact that the interface temperatures are still higher than the aged T-BFRP composite. From Table 3, it can be said that the average temperature for the aged T-BFRP composite was in the range of 29–30 °C respectively throughout the 6.72 km in sliding distance. Therefore, the current work demonstrates a potential way to reduce interfaces temperatures between the composite/counterface (i.e., by aging the T-BFRP composite in their respective solutions with different kinematic viscosity). Through literature survey, it is found that most polymeric composites result in high interface temperatures when applied load increases at longer sliding distance. This is due to the inability of the resinous region to firmly hold the reinforcing elements since themo-mechanical loading is a prime factor. At a brink of risk, gradual deformation of the polymeric composites through rapid material removal from the fibrous and resinous regions will contribute to the high interface temperature.

In summary, betelnut fibres have commercial benefits. From the outcome of the current work, aged T-BFRP composite is superior in tribological performances compared to NP, unaged betelnut fibre composites and CSM-GFRP composite. This can be attributed to the surface roughness of the betelnut fibre due to the presence of *trichomes* which enhance the interlocking of the betelnut fibre in the matrix and the peculiar property of the betelnut fibrous region itself despite the aging period. From the commercial application aspect, aged T-BFRP composite may be considered in producing automotive components as well as bearing and sliding materials. With respect to environmental impact, planting of betelnut trees and harvesting of betelnut fruits are more beneficial to environmental conservation rather than the production of glass fibre. It is vital that due consideration be given to looking at the possibility of commercializing natural fibres that include betelnut fibre as an alternative to glass fibre in the production of polymeric composite materials. However, due to the time limitation of the current study, much work pertaining to the design analysis of the aged T-BFRP composite subjected to the possible proposed applications above remains to be done.

## 4. Conclusions

After performing the experimental works and analyzing the results, the following conclusions can be drawn:

1. For friction performance of the aged T-BFRP composite, both AP-O and P-O showed increasing trends of friction coefficients for the different applied loads (5–30 N) when the kinematic viscosity of the aging solutions increased. This is attributed to the high shear resistance at the rubbing zone between the composite/counterface due to the thickening of the aging solutions at high kinematic viscosity.
2. The friction performance for AP-O and P-O for the aged T-BFRP composite followed the order of R134a > SAE80 > ATF4 > ATF3 > 20W/50 > 15W/50 > 15W/40 > 10W/50 > 10W/40 > 10W/30 > 5W/50 > 5W/40 respectively where T-BFRP composite aged R143a and 5W/40 recorded the highest and lowest values in friction coefficient for AP and P orientations.
3. For the aged NP, there was no sign of obvious fluctuations in friction coefficient profiles for the different aging solutions. Instead, for the same applied load under different aging conditions, aged NP produced the same friction coefficient profiles curves with respect to 6.72 km of sliding distance at 2.8 m/s of counterface sliding velocity.
4. The improvement in friction coefficient for the aged T-BFRP composite in AP_5W/40_ and P_5W/40_ was 32% and 49% as compared to the aged NP for 30 N of applied load. For the same orientation but under different aging condition, i.e., AP_R134a_ and P_R134a_, the improvement in friction coefficient were reduced to 7.5% and 9.9% respectively.
5. On average, the improvement in interface temperature was 45 ± 0.3% for the aged T-BFRP composited in AP and P orientations at 30 N of applied load and 3.32 km of sliding distance as compared to the aged NP.
6. The current work also revealed that at high kinematic viscosity, i.e., high shear resistance of the sliding surfaces, the relative shear resistance incurred by the test samples was much higher than at low kinematic viscosity, i.e., low shear resistance of the sliding surfaces. This increased the interface temperature since the friction component of relative motion of the composite/counterface was high.
7. The wear mechanism for the aged T-BFRP composite at AP-O was predominated by micro-cracks, plastic deformation and debonding of fibres. There was evidence of hollow regions at the resinous regions associated with loose fibre at the worn surfaces.
8. For the aged T-BFRP composite at P-O, the wear mechanism was initiated by adhered fibres parallel to the sliding direction that soon caused fibre debonding resulting in scattered loose fibres and wear debris at the worn surfaces of the composite. Slight fracture at the resinous region was also evidenced due to the absences on the fibre in the polyester matrix.
9. Meanwhile for the aged NP, a large number of micro-cracks which propagated to form macro-cracks and finally fractured the polyester were seen through microscopic analysis. Due to the absence of the fibre and under high loading conditions, large chunks of loose wear debris were seen on the worn surfaces. Moreover, the rate of plastic deformation on the worn samples was severe as compared to AP-O and P-O at 30 N of applied load.
10. On a general note, microscopic images of the worn samples for 30 N of applied load for the aged AP and P orientations revealed that the wear was severe with high absorption rate and less severe for samples with low absorption rate. Hence, it can be said that surface wettability of the aged betelnut fibres worsened when they were aged in solution with low kinematic viscosity.

## Figures and Tables

**Table 1:** Properties of treated betelnut fibre mat.

Length of individual fibres in mat	20–50 mm
Range of the fibre diameters in mat	100–200 μm
Size of fibre mat	100 × 100 mm^2^
Density of fibre mat	200 ± 10 g/m^2^
Average distance of fibres in the mat	83 ± 5 μm
Orientations of fibres in mat	Randomly distributed
Fibre to resin ratio	48 Vol. %

**Table 2:** Kinematic viscosities for the different types of aging solutions.

Solutions	Kinematic Viscosity (cSt) at 100 °C		Ref.
	Minimum	Maximum	
5W/40	3.8/12.5	<16.3	[10]
5W/50	3.8/16.3	<21.9	
10W/30	4.1/9.3	<12.5	
10W/40	4.1//12.5	<16.3	
10W/50	4.1/16.3	<21.9	
15W/40	5.6/12.5	<16.3	
15W/50	5.6/16.3	<21.9	
20W/50	5.6/16.3	<21.9	
SAE80	7.0	<11.0	
ATF D-3	7.2		[11]
ATF D-4	7.5		[12]
R134a	20		[13]

Remark: SAE-Society of Automotive Engineers, ATF-Automatic Transmission Fluid, R134a-type of oil normally used in automotive air conditioning compressors.

**Table 3:** Friction coefficient and interface temperature of the stainless steel counterface for the aged NP and T-BFRP composite as compared to other published works on unaged betelnut fibre and CSM glass fibre composites using POD test method.

Composite Type	Testing Conditions	Average Friction Coefficient; µ	Average Temperature; °C	Ref.
Aged T-BFRP	AL: 30 N SV: 2.8 m/s SD: 6.72 km	AP_5W/40_: 0.6402 ± 0.05 P_5W/40_: 0.4820 ± 0.05	AP_5W/40_: 26 ± 0.3 P_5W/40_: 27 ± 0.3	-
		AP_R134a_: 0.8634 ± 0.03 P_R134a_: 0.8410 ± 0.03	AP_R134a_: 31 ± 0.3 P_R134a_: 30 ± 0.3	
T-BFRP		AP: 0.8214 ± 0.05 P: 0.7431 ± 0.05	AP: 78 ± 0.5 P: 72 ± 0.5	[4,8,16,27,28]
Ut-BFRP		AP: 0.8841 ± 0.05 P: 0.8094 ± 0.05	AP: 50 ± 0.3 P: 53 ± 0.3	[29]
NP	AL: 30 N SV: 2.8 m/s SD: 3.36 km	0.9400 ± 0.05	48–53 ± 0.5	-
CSM-GFRP	AL: 50 N SV: 3.9 m/s SD: 6.72 km	AP: 0.8988 ± 0.05 P: 0.8743 ± 0.05	AP: 85 ± 0.5 P: 82 ± 0.5	[3,30]

Remark: SSCF—stainless steel counterface; ATAC—after testing, after cleaning.
